# Equine trypanosomosis: enigmas and diagnostic challenges

**DOI:** 10.1186/s13071-019-3484-x

**Published:** 2019-05-15

**Authors:** Philippe Büscher, Mary Isabel Gonzatti, Laurent Hébert, Noboru Inoue, Ilaria Pascucci, Achim Schnaufer, Keisuke Suganuma, Louis Touratier, Nick Van Reet

**Affiliations:** 1 Department of Biomedical Sciences, Institute of Tropical Medicine, Nationalestraat 155, 2000 Antwerp, Belgium; 2 Departamento de Biología Celular, Universidad Simón Bolívar, Caracas, 1080 Venezuela; 3 PhEED Unit, Animal Health Laboratory in Normandy, ANSES, 14430 Goustranville, France; 4 Obihiro University of Agriculture and Veterinary Medicine, Obihiro, Hokkaido 080-8555 Japan; 5 Istituto Zooprofilattico Sperimentale dellʼAbruzzo e del Molise “G.Caporale”, Campo Boario, 64100 Teramo, Italy; 6 Centre for Immunity, Infection and Evolution, Institute of Immunology and Infection Research, University of Edinburgh, Charlotte Auerbach Road, Edinburgh, EH9 3FL UK; 7 Consultant member of the OIE Non-Tsetse Transmitted Animal Trypanosomoses Network, Bordeaux, France

**Keywords:** Equine, Dourine, Nagana, Surra, Diagnosis, *Trypanosoma brucei*, *Trypanosoma congolense*, *Trypanosoma equiperdum*, *Trypanosoma evansi*, *Trypanosoma vivax*

## Abstract

Equine trypanosomosis is a complex of infectious diseases called dourine, nagana and surra. It is caused by several species of the genus *Trypanosoma* that are transmitted cyclically by tsetse flies, mechanically by other haematophagous flies, or sexually. *Trypanosoma congolense* (subgenus *Nannomonas*) and *T. vivax* (subgenus *Dutonella*) are genetically and morphologically distinct from *T. brucei*, *T. equiperdum* and *T. evansi* (subgenus *Trypanozoon*). It remains controversial whether the three latter taxa should be considered distinct species. Recent outbreaks of surra and dourine in Europe illustrate the risk and consequences of importation of equine trypanosomosis with infected animals into non-endemic countries. Knowledge on the epidemiological situation is fragmentary since many endemic countries do not report the diseases to the World Organisation for Animal Health, OIE. Other major obstacles to the control of equine trypanosomosis are the lack of vaccines, the inability of drugs to cure the neurological stage of the disease, the inconsistent case definition and the limitations of current diagnostics. Especially in view of the ever-increasing movement of horses around the globe, there is not only the obvious need for reliable curative and prophylactic drugs but also for accurate diagnostic tests and algorithms. Unfortunately, clinical signs are not pathognomonic, parasitological tests are not sufficiently sensitive, serological tests miss sensitivity or specificity, and molecular tests cannot distinguish the taxa within the *Trypanozoon* subgenus. To address the limitations of the current diagnostics for equine trypanosomosis, we recommend studies into improved molecular and serological tests with the highest possible sensitivity and specificity. We realise that this is an ambitious goal, but it is dictated by needs at the point of care. However, depending on available treatment options, it may not always be necessary to identify which trypanosome taxon is responsible for a given infection.

## Background

Equine trypanosomosis is a complex of infectious diseases called dourine, nagana and surra that are caused by several closely related species of *Trypanosoma*. They give rise to important economic losses in Africa, the Middle East, Asia and Latin America. Nevertheless, they can be considered as animal diseases that are seriously neglected, both by the scientific community as by veterinary authorities and regulatory organisations. The situation is aggravated by the reluctance of many endemic countries to notify dourine and surra to the World Organisation for Animal Health. Major obstacles to the local and global control of equine trypanosomosis are the lack of vaccines, the inability of drugs to cure the neurological stage of the infections, the inconsistent case definitions and the limitations of current diagnostics. Recent outbreaks of surra and dourine in Europe illustrate the risk and consequences of importation of equine trypanosomosis with infected animals into non-endemic countries. In view of the ever-increasing movement of horses around the globe, there is not only the obvious need for reliable curative and prophylactic drugs but also for accurate diagnostic tests and algorithms. The aim of this article is to provide an overview of the existing knowledge on the aetiology of equine trypanosomosis and the taxonomic status of the infecting trypanosomes, the geographical distribution, prevention and treatment options and diagnostic tools. We further discuss the issue of imperfect case definitions and propose research to address the limitations of current diagnostics.

## Aetiology

Equine trypanosomosis is an infectious disease that is caused by several species of the genus *Trypanosoma*, including *T. evansi*, *T. equiperdum*, *T. brucei, T. vivax, T. congolense* and *T. cruzi.* Infections of horses with *T. cruzi* are very rare and are not further considered here [1]. Historically, the diseases caused by these trypanosomes are called “surra” (*T. evansi*), “dourine” (*T. equiperdum*) and “nagana” (*T. brucei*, *T. congolense* and *T. vivax*) but careful examination of published and unpublished data reveals that for all these three diseases, the clinical signs observed, including ventral oedema, emaciation, anaemia and neurological symptoms, can be very similar and are certainly not pathognomonic [2–7].

## Transmission and resulting geographical distribution

*Trypanosoma brucei* and *T. congolense* are the only species that are confined to the distribution of tsetse flies (the vector) in sub-Saharan Africa. *Trypanosoma equiperdum* is transmitted sexually, and *T. evansi* is transmitted mechanically by blood-sucking flies, vampire bats, and possibly sexually [8, 9].

Oral transmission of *T. evansi via* contaminated meat or carcasses is well documented but normally does not occur in equines [10, 11]. *Trypanosoma vivax* can be transmitted both cyclically by tsetse flies and mechanically by other haematophagous flies. The global distribution of *T. equiperdum, T. evansi* and *T. vivax* is much wider, including Africa and Latin America for *T. vivax*, Africa, Latin America and Asia with sporadic import cases in Europe for *T. evansi* and worldwide, except Oceania, for *T. equiperdum* [12, 13]. Most countries where trypanosomoses are endemic do not regularly report on these diseases, and as a consequence, the exact burden and area of distribution remain largely unknown. For example, a systematic review on surra shows serious discrepancy between countries reporting this disease to the World Organisation for Animal Health (OIE) and countries for which published evidence of surra exist [12]. From recent reviews on surra it becomes clear that its distribution map is based on anecdotal observations [12–15]. No such recent reviews exist on *T. brucei*, *T. congolense*, *T. equiperdum* and *T. vivax* in horses. However, evidence is increasingly being published on horses infected with *T. evansi*, *T. equiperdum* and *T. vivax* in Brazil, Ethiopia, India, Israel, Jordan, Mongolia, Nigeria, Pakistan, South East Asia, Sudan, Venezuela, etc. [12, 13, 16–25].

## Prevention and treatment

Vaccines against equine trypanosomosis do not exist. Chemotherapy of equine trypanosomosis consists of treatment with diminazene diaceturate, isometamidium chloride, quinapyramine chloride/quinapyramine sulphate combination, suramin or melarsomine hydrochloride. Except for trypanosome strains that display an innate or acquired resistance, these drugs are able to clear the parasites from the blood circulation [26]. However, except for *T. congolense*, all the other trypanosomes are known to reside mainly in extravascular spaces of many tissues and organs, including the central nervous system. Evidence is accumulating that none of the aforementioned drugs is effective in the neurological stage of the disease since none is able to cross the blood-brain-barrier in sufficient amounts [24, 27–30]. Still, a recent outbreak of surra (caused by *T. evansi*) in Spain was brought under control upon treatment with melarsomine hydrochloride (Cymelarsan, Merial, France) of all parasitologically confirmed and suspect animals (dromedary camels, horses, donkeys) as well as of all animals that were in direct or indirect contact with the index case [31]. The latter was a dromedary camel imported from Gran Canaria, without prior screening for surra, one-and-a-half years before the disease was detected [31, 32]. Another outbreak, this time of dourine (caused by *T. equiperdum*), occurred in Italy in 2011 and illustrates the risk of importation of equine trypanosomosis with infected animals into non-endemic countries [33]. That outbreak was only brought under control thanks to drastic measures taken by the veterinary authorities over several years [6, 33, 34]. A potential, yet undocumented, risk of importation of equine trypanosomosis into non-endemic countries is by artificial insemination with contaminated semen [7].

## Taxonomy and morphology

*Trypanosoma congolense* (subgenus *Nannomonas*) and *T. vivax* (subgenus *Duttonella*) are species that are clearly separated, both genetically and morphologically, from the other taxa within the genus *Trypanosoma*. On the other hand, whether *T. brucei*, *T. equiperdum* and *T. evansi*, traditionally grouped together under the subgenus *Trypanozoon*, can be considered distinct species remains controversial. Morphologically, *T. brucei* can be distinguished from the latter two taxa by its pleomorphic nature with long slender, intermediate and short stumpy trypomastigote forms present in the mammalian host. The short stumpy forms are those that will initiate infection in the vector of nagana, the tsetse fly [5]. *Trypanosoma equiperdum* and *T. evansi* are monomorphic and display only long slender forms, although in *T. evansi*, intermediate forms may sometimes be found [35]. Lack of monophyly of *T. equiperdum* and *T. evansi*, and the fact that genetic differences to *T. brucei* are small, have led to proposals that they should be classified as subspecies or strains of *T. brucei* [35–38]. Other studies have proposed that many so-called *T. equiperdum* isolates are in fact misidentified *T. evansi* strains, and that *T. evansi* and *T. equiperdum* evolved separately and on more than one occasion from *T. brucei* [38–41]. Today, based on kinetoplast minicircle differences, *T. evansi* is further divided into *T. evansi* type A and type B, the latter so far only isolated from camels in eastern Africa [42]. The “true” (i.e. non-controversial) *T. equiperdum* strains can be divided in at least two and maybe more clades of distinct evolutionary origin [38, 41]. Newly isolated *T. equiperdum* strains from outbreaks in Italy and in Mongolia may even further complicate the taxonomic situation [6, 16]. Table 1 summarises the major characteristics of *Trypanosoma* taxa that may cause trypanosomosis in equines.

**Table 1:** Overview of some characteristics of different taxa within the genus *Trypanosom*a causing equine trypanosomosis

	*Trypanozoon*			*Duttonella*	*Nannomonas*
	*T. brucei*	*T. evansi* type A	*T. equiperdum*	*T. vivax*	*T. congolense*
Distribution	Sub-Saharan Africa	Africa, Latin America, Middle East, Asia	Worldwide except Oceania USA and Canada	Africa, Latin America	Sub-Saharan Africa
Mammalian host range	Multi-species	Multi-species	Equines	Multi-species	Multi-species
Transmission	Tsetse	Biting flies, vampire bats, orally (sexually?)	Sexually (orally *via* milk?)	Tsetse, biting flies	Tsetse
Morphology in the mammalian host	Pleomorphic^a^	Monomorphic^b^	Monomorphic	Monomorphic	Monomorphic
Kinetoplast	Complete	Dyskinetoplastic^c^ or akinetoplastic^d^	Dyskinetoplastic	Complete or dyskinetoplastic	Complete
Kinetoplast minicircle type		A	C or undefined		
ATPase γ C-terminus mutations		A281-	A273P or WT		

^a^Pleomorphic: present as long slender, short stumpy and intermediate trypomastigotes during an infection

^b^Monomorphic: long slender trypomastigotes with anecdotal evidence for partial pleomorphism in *T. evansi* [35]

^c^Dyskinetoplastic: partial loss of kinetoplast DNA, in particular maxicircle DNA

^d^Akinetoplastic: complete lack of kinetoplast DNA

## Diagnosis

Diagnosis of equine trypanosomosis can be challenging due to the absence of specific clinical signs, and because parasitaemias in infected hosts are usually below the detection limit of parasitological tests and can even be below the detection limit of molecular DNA tests [33]. Therefore, diagnosis heavily relies on the combination of clinical signs, serological evidence of infection and epidemiological context. Importantly, where several species co-exist, mixed infections may be frequent [43, 44].

## Clinical diagnosis

Clinical signs of nagana, dourine and surra vary with disease stage (acute, chronic, neurological stage), infecting strain and variable intra- and inter-species susceptibility of the host.

For example, donkeys can carry an infection without developing classical signs of the diseases [45]. Typical, but not pathognomonic, are pyrexia, anaemia, ventral and genital oedema, urticarial plaques, conjunctivitis and keratitis, etc. [5, 28, 46–48]. In the neurological phase of infection with *T. brucei*, *T. equiperdum* or *T. evansi*, ataxia and paralysis of the hind quarter and lips usually precede death. The clinical course of the disease and long-term outcome depend on the parasite strain involved and the immunological status of the host. Other equine diseases that share some clinical signs with trypanosomosis are equine viral arteritis, equine infectious anaemia, contagious equine metritis, anthrax [5].

## Parasitological diagnosis

For microscopic parasitological diagnosis, the most common biological fluid examined is blood, but parasites may be detected in lymph aspirated from superficial lymph nodes, cerebrospinal fluid, milk and vaginal or preputial discharges [6, 16, 49, 50]. In principle, it is possible to distinguish *T. vivax* and *T. congolense* from the *Trypanozoon* species based on morphology, but this is not evident for the species within the subgenus *Trypanozoon*. In case of a microscopic trypanosome-positive specimen, species identification is best performed with a combination of molecular tests that have also the advantage of having a lower detection limit than microscopic parasite detection and that are able to detect mixed infections that may remain cryptic by microscopic examination [43].

## Molecular diagnosis

A plethora of molecular diagnostic tests has been described which target DNA sequences that are specific at different taxonomic levels and which use diverse technology. The most commonly used test formats are polymerase chain reaction (PCR) and quantitative PCR (qPCR). The *18S* and ITS1 sequences within the ribosomal DNA (rDNA) locus as well as satellite DNA are preferential targets for species-specific molecular diagnostics because of their multi-copy nature and the possibility to discriminate the taxa of the subgenus *Trypanozoon* from *T. congolense* and *T. vivax* [16, 51–54]. Distinguishing the taxa within *Trypanozoon* is more challenging. PCR tests based on the mitochondrial DNA of these parasites (kinetoplast DNA or kDNA) can potentially distinguish *T. evansi* and some *T. equiperdum* from *T. brucei* by the absence of the maxicircle component of kDNA, and identify *T. evansi* type B by presence of the type B minicircle [42, 55, 56]. However, many *T. evansi* strains are naturally akinetoplastic (i.e. complete lack of kDNA) so that molecular tests targeting kDNA have limited value. Some authors claim that *T. evansi* type A can be identified by the presence of the type A-specific RoTat 1.2 variant surface glycoprotein (VSG) gene and *T. evansi* type B by the presence of the type B-specific JN 2118HU VSG [57–59]. However, both are single-copy genes which inherently limits the analytical sensitivity of PCRs based on these VSGs and their absence in the genome of *T. brucei* has not been investigated extensively. Furthermore, the VSG repertoire is subject to recombination and therefore inherently unstable, and other researchers have shown that some Kenyan *Trypanozoon* isolates with type A minicircles appear to lack the RoTat 1.2 gene, while the JN 2118Hu VSG gene was also found in *T. b. gambiense* type II [56, 60]. Unfortunately, today there is no simple molecular test that is able to distinguish *T. brucei* from *T. equiperdum*, which means that the epidemiological context has to be taken into account to identify the trypanosome species in an infected horse.

## Serological diagnosis

In contrast to *T. congolense* and, to a lesser extent, *T. vivax*, *Trypanozoon* trypanosomes are primarily tissue parasites; parasitaemia is often very low and seldom reaches the threshold of current parasitological or even molecular diagnostic tests, especially in asymptomatic carriers. Therefore, antibody detection tests are available and can provide indirect evidence of infection.

The complement fixation test (CFT) and the indirect fluorescence antibody test (IFAT) are the only OIE recommended tests for *T. equiperdum* infection [61]. For *T. evansi* infection, OIE recommends IFAT, an enzyme linked immunosorbent assay (ELISA/RoTat 1.2), a card agglutination test for trypanosomosis using *T. evansi* antigen (CATT/*T. evansi*) and immune trypanolysis (IT) [62]. For infections with *T. brucei*, *T. vivax* and *T. congolense*, OIE recommends using IFAT and ELISA [63].

The IT test detects exclusively antibodies that recognise one single multi-copy epitope at the surface of the RoTat 1.2 VSG expressing *T. evansi* type A [64]. As a result, the specificity of IT test is extremely high, but the disadvantages of the test are its complexity and its inability to detect antibodies against other trypanosomes. The CATT/*T. evansi* test is a direct agglutination test that makes use of the same *T. evansi* RoTat 1.2 clone used in the IT test, but the preparation of the reagent exposes other surface antigens as well, resulting in some cross-reactivity with *T. vivax* and *T. equiperdum* [49, 65, 66]. Lower cross-reactivity is expected in the ELISA/RoTat 1.2 with purified VSG RoTat 1.2 as antigen.

Other serological tests use crude preparations derived from bloodstream form trypomastigotes grown *in vivo* (mice or rats) or propagated *in vitro* [67, 68]. The CFT test for dourine, using whole cell extracts from a *T. equiperdum* strain, has the advantage that it, to a certain level, also reacts with antibodies against *T. evansi*; however, this also entails the risk of false positive reactions [67]. Since *T. equiperdum* infection is considered non-curable, any false positive reaction will have serious consequences (castration, isolation, slaughtering of CFT positive animals). Reactivity of CFT with horses infected with *T. brucei,* or even with *T. congolense* or *T. vivax*, seems possible but remains undocumented. An additional problem with CFT is that reactivity may vary in function of the trypanosome strain used to prepare the antigen and of the VSG expressed by the trypanosomes at the moment of harvesting. Efforts to harmonise the antigen preparation of CFT in the diverse laboratories around the world are challenged by the fact that, within the subgenus *Trypanozoon*, genetic distinction between *T. equiperdum*, *T. evansi* and *T. brucei* is blurred, as discussed above [38, 40, 41, 60, 69, 70]. Among scientist members of the OIE Non-Tsetse Transmitted Animal Trypanosomoses Network [71], the consensus is that the Onderstepoort Veterinary Institute (OVI) strain can be adopted as reference strain for *T. equiperdum* for the purpose of CFT antigen preparation. It is this strain that is now used by the European Reference Laboratory for Equine Diseases in France, the National Reference Laboratory for Dourine in Germany and the USDA National Veterinary Services Laboratories [67, 68]. New Mongolian *T. equiperdum* isolates have not been fully typed; whether they may serve as reference strains for the Asian region remains to be investigated [16].

Similar to the CFT, the trypanosome strains used for IFAT and ELISA for *T. evansi* (other than RoTat 1.2), *T. brucei*, *T. vivax* and *T. congolense* have not been defined. Furthermore, as a result of antigenic variation, it is not possible to control the particular VSG that is expressed at the moment of antigen preparation. Since this VSG accounts for 10% of the protein content of a bloodstream form trypomastigote, it is a major, yet undefined, component of crude antigen preparations for IFAT and ELISA. Other proteins in crude trypanosome antigen preparations may cross-react with antibodies unrelated to infection with trypanosomes [72].

## Inconsistent case definitions

Case definitions with unequivocal criteria to identify a case of dourine, surra and nagana are essential to estimate the impact of these diseases on the horse population, to guide surveillance, control and prevention strategies, and to choose among treatment options, the latter taking into account that treatment success is largely dependent on the stage of the diseases rather than on the infecting trypanosome strain. An attempt at such case definition was made during the dourine outbreak in Italy in 2011; subsequently, the same case definition was adopted by the OIE Terrestrial Manual [6, 33]. According to this definition, a case of dourine is considered confirmed when an animal has a positive result with CFT, IFA or PCR, and (i) shows clinical signs compatible with dourine; (ii) shows an increase in serological CFT titre in two consecutive tests; or (iii) is epidemiologically linked with a confirmed case of dourine [33, 61, 73]. An important limitation to consider here is that this case definition only fully applies if (i) dourine as a disease is exclusively caused by *Trypanozoon* parasites that can be reliably distinguished from *T. brucei* and *T. evansi* and (ii) if CFT, IFAT and PCR exist that are fully specific for these parasites. Unfortunately, both conditions are not fulfilled.

According to the OIE Terrestrial Manual [62], an equid is negative to surra if it is negative to ELISA-*T. evansi* (anti-horse IgG whole molecule), CATT/*T. evansi*, PCR-TBR (i.e. PCR targeting *Trypanozoon*-specific satellite DNA [54]) and microscopic examination. An equid is considered infected with *Trypanozoon spp.* (*sic*) if it is positive to PCR-TBR and/or if *Trypanozoon* parasites are observed by microscopic examination. An equid is considered as seropositive to surra if it is positive to ELISA-*T. evansi* and/or CATT/*T. evansi*; in this case, the animal should be tested for CFT-dourine, and, if it is positive for CFT-dourine, it is also considered as seropositive to dourine; if it is negative to CFT-dourine, it is considered as seropositive to surra only [62]. This “composite” case definition takes into account inherent difficulties to distinguish the *Trypanozoon* taxa, but the definition of seropositivity is questionable and does not take into account possible cross-reactions with *T. brucei*, *T. vivax* and *T. congolense*. It also disregards the possibility to use more specific tests such as the IT and the ELISA/RoTat 1.2 to rule out or suggest infection with *T. evansi* type A. Both may miss *T. evansi* type B infections, but *T. evansi* type B has so far only been isolated from dromedary camels in Kenya and Ethiopia [55, 56].

For equine infections with *T. brucei*, *T. vivax* and *T. congolense*, the OIE Terrestrial Manual does not provide any case definition.

## How can the limited specificity of current diagnostics for equine trypanosomosis that impedes international movement of competition horses be addressed?

In order to improve the specificity of diagnostics for equine trypanosomosis, the focus should be on molecular and serological tests.

Molecular tests can identify with relatively high sensitivity and excellent specificity *T. congolense*, *T. vivax* and *Trypanozoon* taxa. However, within *Trypanozoon*, no single test is able to identify each taxon unequivocally. On the other hand, for the purpose of international movement of horses, it seems sufficient to use a combination of genus- and subgenus-specific molecular tests. As qPCR is becoming the standard now, the conventional PCR tests might be transformed into qPCR to improve standardisation and sensitivity. In any case, given the usually low parasitaemia in clinically healthy, yet infected, equines, the negative predictive value of molecular tests is inherently low.

Regarding serological tests, improvements are possible with respect to test format and antigens. CFT and IFAT should be replaced by techniques that are less complex and easier to standardise, such as ELISA for high throughput or immunochromatography for individual testing. Important to keep in mind, however, is the market failure that prevents commercial companies to invest in the development of such tests. The diagnostic specificity of antibody detection tests is largely defined by the antigen preparation. Therefore, the use of crude antigen preparations should be discouraged, and initiatives to replace them with recombinant or synthetic peptides should be supported. For *T. congolense* and *T. vivax*, recombinant fragments of respectively Cathepsin B1 and the cytoskeleton associated protein GM6 are used in the VeryDiag test [74]. Also, recombinant *T. vivax* Cathepsin L has some diagnostic potential [75]. For the species of subgenus *Trypanozoon*, many common proteins have already been expressed as recombinant antigens with proven diagnostic potential, in particular the invariant surface glycoproteins 65 and 75 (ISG65, ISG75), the cytoskeleton associated protein GM6 and, more recently, a *T. equiperdum* protein that exhibits homology with the regulatory subunit of mammalian cAMP-dependent protein kinases [76–86]. None of them, however, have been evaluated for their diagnostic accuracy in *Trypanozoon* infections in general, irrespective of the infecting trypanosome species and geographical origin. In case there is a need to detect *T. evansi* type A specific antibodies, a recombinant fragment of VSG RoTat 1.2, expressed in different systems, has shown its diagnostic potential [80, 87–89].

## Conclusions

To address the limitations of the current diagnostics for equine trypanosomosis we recommend not to aim for the distinction between taxa within the subgenus *Trypanozoon*. We propose to conduct studies into (i) improved molecular tests with the highest possible sensitivity; and (ii) improved serological tests with the highest possible specificity. For the proper evaluation of these diagnostics, it will be necessary to establish a panel of representative trypanosome strains, and their corresponding DNA/RNA, as well as a panel of biological specimens (serum/plasma/blood) from horses naturally or experimentally infected with *T. vivax*, *T. congolense* and representative *Trypanozoon* taxa.

## Abbreviations

18S: 18 Svedberg unit
ATP: adenosine triphosphate
cAMP: cyclic adenosine monophosphate
CATT: card agglutination test for trypanosomiasis
CFT: complement fixation test
IFAT: indirect fluorescence antibody test
IgG: immunoglobulin G
ISG: invariant surface glycoprotein
ITS1: internal transcribed spacer 1
kDNA: kinetoplast DNA
OIE: Office International des Epizooties, World Organisation for Animal Health
OVI: Onderstepoort Veterinary Institute
PCR: polymerase chain reaction
qPCR: quantitative PCR
rDNA: ribosomal DNA
RNA: ribonucleic acid
RoTat: Rhode *Trypanosoma* antigen type
TBR: *Trypanosoma brucei* repetitive satellite DNA sequence
USDA: United States Department of Agriculture
VSG: variant surface glycoprotein
WT: wild type
